# QuickStats

**Published:** 2013-06-14

**Authors:** Patricia F. Adams, Michael E. Martinez

**Figure:**
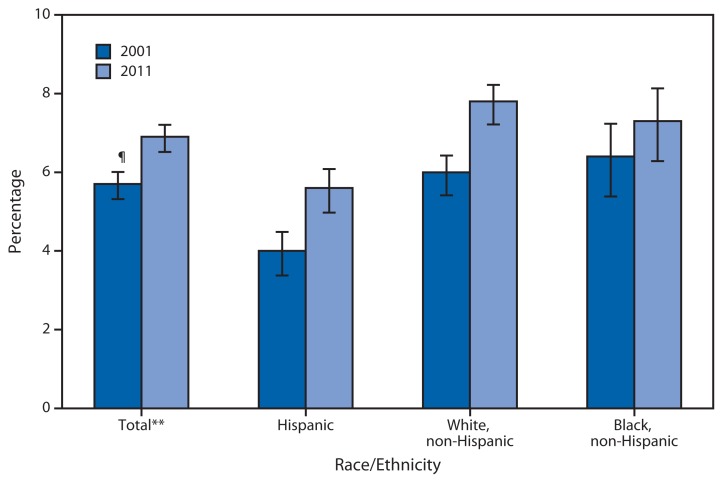
Percentage of Persons Aged <18 Years Who Received Special Educational or Early Intervention Services,^*^ by Race/Ethnicity^†^ — National Health Interview Survey, United States, 2001 and 2011^§^ ^*^ Based on response to the question, “Do any of the following [family members aged <18 years] receive special educational or early intervention services?” Special educational and early intervention services are designed to meet the needs of a child with special needs or disabilities and are provided by the state or school system at no cost to the parent. Early intervention services might include, but are not limited to, medical and social services, parental counseling, and therapy. ^†^Persons of Hispanic ethnicity might be of any race or combination of races. ^§^Estimates are based on household interviews of a sample of the civilian noninstitutionalized U.S. population and are derived from the National Health Interview Survey Family Core component. ^¶^95% confidence interval. ^**^Includes other races not shown separately.

From 2001 to 2011, the percentage of children aged <18 years who were receiving special educational or early intervention services increased overall and among Hispanic and non-Hispanic white children, no change was observed among non-Hispanic black children. In 2001 and 2011, Hispanic children were less likely than non-Hispanic white and non-Hispanic black children to receive these services.

**Sources:** Barnes PM, Adams PF, Schiller JS. Summary health statistics for the U.S. population: National Health Interview Survey, 2001. Vital Health Stat 2003;10(217). Available at http://www.cdc.gov/nchs/data/series/sr_10/sr10_217.pdf.

Adams PF, Kirzinger WK, Martinez ME. Summary health statistics for the U.S. population: National Health Interview Survey, 2011. Vital Health Stat 2012;10(255). Available at http://www.cdc.gov/nchs/data/series/sr_10/sr10_255.pdf.

